# A Case of Mpox Reinfection

**DOI:** 10.1093/cid/ciad147

**Published:** 2023-03-11

**Authors:** Stefano Musumeci, Iris Najjar, Emmanuelle Boffi El Amari, Manuel Schibler, Frédérique Jacquerioz, Sabine Yerly, Adriana Renzoni, Alexandra Calmy, Laurent Kaiser

**Affiliations:** HIV/AIDS Unit, Division of Infectious Diseases, Geneva, Switzerland; Division of Infectious Diseases, Geneva, Switzerland; Geneva Center for Emerging Viral Diseases, Geneva University Hospitals, Geneva, Switzerland; Emmanuelle Boffi El Amari Private Practice, Geneva, Switzerland; Division of Infectious Diseases, Geneva, Switzerland; Laboratory of Virology, Division of Laboratory Medicine, Geneva, Switzerland; Geneva Center for Emerging Viral Diseases, Geneva University Hospitals, Geneva, Switzerland; Division of Tropical and Humanitarian Medicine, Geneva, Switzerland; Primary Care Division, Geneva University Hospitals, Geneva, Switzerland; Laboratory of Virology, Division of Laboratory Medicine, Geneva, Switzerland; Laboratory of Virology, Division of Laboratory Medicine, Geneva, Switzerland; HIV/AIDS Unit, Division of Infectious Diseases, Geneva, Switzerland; Division of Infectious Diseases, Geneva, Switzerland; Geneva Center for Emerging Viral Diseases, Geneva University Hospitals, Geneva, Switzerland; Laboratory of Virology, Division of Laboratory Medicine, Geneva, Switzerland

**Keywords:** mpox, *Monkeypox virus*, immunity, reinfection

## Abstract

A healthy young man first diagnosed with mpox in May 2022 presented again in November 2022 with anal proctitis and a positive polymerase chain reaction on a rectal swab for *Monkeypox virus* after a recent trip to Brazil, where he engaged in condomless sexual intercourse with multiple male partners.

## Graphical abstract

**Graphical abstract:**
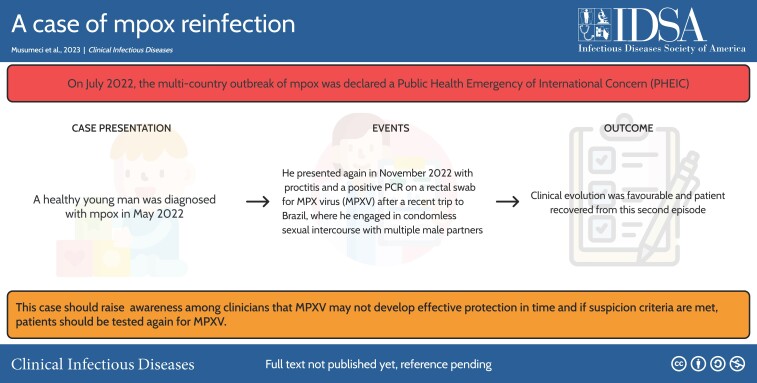
A graphical abstract is available at Tidbit: https://tidbitapp.io/tidbits/a-case-of-mpox-reinfection-21dcc2d0-7dff-4888-bd8c-823af17756df.

Mpox, caused by *Monkeypox virus* (MPXV), was considered a sporadic zoonotic disease limited to West and Central African countries until the current worldwide outbreak was declared in May 2022. MPXV belongs to the *Orthopoxvirus* genus, as do *Vaccinia virus*, *Cowpox virus*, and *Variola virus*, all of which are infectious to humans. MPXV infection generates humoral and cellular immunity that is expected to provide long-term protection against reinfection [1]. These assumptions are based on extensive experience with *Variola virus*, the agent of smallpox disease [2]. Smallpox infection or vaccination with the attenuated *Vaccinia virus* provided almost complete protection from infection with *Variola virus* thanks to cross-immunity [2]. Even years after the last smallpox vaccination, individuals historically vaccinated are considered less likely to be infected by MPXV than nonvaccinated persons and protected from severe disease [3]. MPXV reinfection has only been reported by Golden et al [4], so far suggesting it is at most an infrequent event. Since 1980, waning immunity against smallpox of historically vaccinated or naturally infected persons and the heightened number of susceptible individuals have been considered the main drivers for the mpox flares in African endemic regions. In the Democratic Republic of Congo, between 1980 and 2007, reported infections with MPXV increased 20-fold. While these studies suggest protective, long-lasting cross-immunity through smallpox vaccination, data on elicited protection after MPXV infection are scarce. Here we report a case of *Monkeypox virus* reinfection in an otherwise healthy adult.

## CASE REPORT

A 34-year-old man in good general health presented in May 2022 with 4 umbilicated lesions on the penis. He was on preexposure prophylaxis for human immunodeficiency virus (HIV) and reported having had condomless sex with men, including receptive anal intercourse. He did not report previous smallpox infection or vaccination. On physical examination, no lymphadenopathy was found, and oropharyngeal and anal inspection was normal. The patient did not report fever or any other symptoms. Skin lesions and pharyngeal swabs tested positive for MPXV by real-time *Orthopoxvirus* polymerase chain reaction (PCR) [5] with cycle threshold (Ct) values of 16.5 and 35.3, respectively. He was also diagnosed with asymptomatic urinary *Chlamydia trachomatis* infection. Within 2 weeks, skin lesions spontaneously resolved without complications.

On 1 December 2022, the patient reported persistent perianal pain without bleeding nor discharge. Symptoms had started 2 weeks before, on his return from Brazil in November 2022, where he had engaged in condomless anal intercourse with multiple male partners. No other complaints or symptoms were reported. He related a last receptive anal intercourse 2 weeks before the presentation. The patient did not recall any signs or symptoms of mpox among his partners, nor was he aware of an MPXV infection among them. Clinical examination found no skin lesion in the perianal region or other body parts. Apart from a slightly palpable painless right inguinal lymphadenopathy, the medical examination was normal. Four weeks after symptom onset, a proctologic examination revealed a small anal fissure but no typical mpox lesion.

Sexually transmitted infectious diseases were screened using standard diagnostic tests. The anal swab done on 1 December 2022 came back positive for MPXV with a Ct of 27 and for *C. trachomatis* (non–lymphogranuloma venereum). The latter was treated with a 7-day course of doxycycline. HIV serology and viremia were negative, and syphilis was serology negative for acute infection. On 13 December 2022, rectal and pharyngeal swabs were collected again and turned out negative. *Orthopoxvirus* PCR was also negative in the blood and urine on 13 December 2022. Total MPXV antibodies were tested (Custom Monkeypox Human ELISA Kit, RayVio, Georgia) on sera collected prior to the first episode in May 2022 and after the second in December 2022: they came back negative and positive, respectively. Unfortunately, no serum had been collected between the 2 episodes.

## DISCUSSION

We report a case of probable MPXV reinfection. The patient's *Orthopoxvirus* PCR returned positive on an anal swab 14 days after the last receptive anal intercourse and 6 months after a previously confirmed MPXV infection. It is not possible to determine if this second mpox episode contributed to the proctitis in the context of concomitant *C. trachomatis* infection and the presence of an anal fissure. Specific mpox mucosal lesions might have been missed as the proctologic examination was carried out 4 weeks after symptom onset. It is therefore impossible to distinguish between symptomatic and asymptomatic reinfection. Though viral culture could not be performed to confirm active infection with certainty, a trusted physician’s detailed anamnesis undertaken during the second episode makes anal contamination following condomless anal intercourse with an MPVX-positive partner in the days before sampling unlikely. The disease course was mild, and the patient recovered completely. No immunosuppressive condition were found and HIV could be excluded.

This case illustrates that sterilizing immunity may be limited despite prior systemic infection. Studies suggest that historical smallpox infection or vaccination produces cross-immunity against MPXV [3, 6]. The vaccine currently used against MPXV, the Modified Vaccinia Ankara vaccine, an attenuated live nonreplicating vaccinia virus in human cells, was shown to elicit sufficient cross-neutralizing activity against MPXV to prevent infection and reduce disease severity [7]. After MPXV infection, a study showed that 83% and 71% of historically vaccinated and nonvaccinated individuals developed MPXV-neutralizing antibodies in serum [6]. Of these, neutralization antibody titers were low irrespective of vaccination status [6]. One possible explanation given by the authors was the sampling time, which occurred early in the symptomatic phase [6]. These findings might also be explained by the not well-established neutralization assays used for MPXV, possibly leading to lower sensitivities. Nonetheless, natural MPXV infection may not elicit sufficient neutralizing antibody responses in a subset of healthy individuals. However, neutralization activity most likely only partially reflects MPXV elicited immune response as cellular immunity was also determined to be important [8]. In addition, the role of mucosal immunity remains unclear; the same holds for whether the primary site of MPXV infection—here, the genitals—elicits sufficient mucosal immunity on every other mucosa, such as the gastrointestinal tract. Indeed, mucosal immunity was shown to be critical in mucosal-transmitted viral infections [9].

Reinfection in our patient most likely occurred in Brazil, which in November 2022 had one of the highest reported daily new cases of mpox. Because of the nature of MPXV, a double-stranded DNA virus that has shown few variations since the outbreak's beginning, it seems unlikely that reinfection occurred because of a mutation-driven immune escape of a Brazilian variant. The patient's first isolate was sequenced confirming MPXV B.1., but a technical issue and lack of sufficient specimen precluded obtaining the second sequence (Oxford Nanopore MinION), so strain comparison could not be made. With only 2 Brazilian MPXV sequences reported on Nextstrain, the hypothesis of a mutation-driven immune escape cannot be excluded entirely. Yet, the large cross-immunity between different orthopoxviruses and the large number of shared epitopes driving the immune response [10] make the appearance of an immune escape mutation that would impede the activity of cross-reactive antibodies unlikely. Nevertheless, numerous immune evasion mechanisms have been described in poxviruses, mainly in the better-studied *Vaccinia virus* [11] but also in MPXV, which was shown to inhibit T-cell activation [12]. Yet, the clinical significance is not well understood.

In this case, the high rate of mucosal lesions associated with anal intercourse leading to high viral load exposure might have outweighed the host's local immune response and led to local and transient replication of MPXV. Asymptomatic shedding of MPXV in MPXV-naive individuals has previously been described [13]. In MPXV-nonnaive individuals, asymptomatic reinfection and shedding might also occur, particularly in the case of large inocula and because a subset of otherwise healthy individuals may not develop sterilizing immunity. This could have major public health implications as it could lead to a rise of asymptomatic or paucisymptomatic MPXV reinfections, possibly leading to a rise in symptomatic mpox in naive individuals. This would ineluctably rise the question of extending vaccination to recovered individuals.

Furthermore, persistent MPXV-positive PCR results after infection have been reported in the literature and are suggestive for prolonged MPXV shedding [14]. Our patient was tested positive for MPXV 6 months after his first episode, and a repeated rectal swab 13 days after the second diagnosis came back negative. A mathematical modeling study found a median time of rectal sample PCR positivity of 8.3 days with a 95th percentile at 14.7 days [15]. To our knowledge, persistent shedding over this time frame has not been reported so far, regardless of the sampling site, making the hypothesis of persistent shedding from his first infection unlikely.

## CONCLUSIONS

While the next few months will be decisive in determining whether this case is an exception or the first of a beginning series, it is crucial to raise awareness among clinicians that MPXV may not develop effective protection in time and that if suspicion criteria are met, patients should be tested again for MPXV. With the growing evidence for asymptomatic and/or prolonged shedding of MPXV, screening among naive, recovered, and vaccinated individuals also needs to be addressed. Meanwhile, public health authorities should be informed and prepare for the consequences that increasing MPXV reinfections would imply for the management of the current mpox epidemic. Further tools may be useful, such as the development of specific MPXV vaccines.

## Notes

***Acknowledgments.*** The authors thank Florian Laubscher for the help on the viral sequencing process.
